# Artificial Intelligence-Based Thyroid Nodule Classification Using Information from Spatial and Frequency Domains

**DOI:** 10.3390/jcm8111976

**Published:** 2019-11-14

**Authors:** Dat Tien Nguyen, Tuyen Danh Pham, Ganbayar Batchuluun, Hyo Sik Yoon, Kang Ryoung Park

**Affiliations:** Division of Electronics and Electrical Engineering, Dongguk University, 30 Pildong-ro 1-gil, Jung-gu, Seoul 04620, Korea; nguyentiendat@dongguk.edu (D.T.N.); phamdanhtuyen@gmail.com (T.D.P.); ganabata87@gmail.com (G.B.); yoonhs@dongguk.edu (H.S.Y.)

**Keywords:** artificial intelligence, thyroid nodule classification, deep learning, Fast Fourier transform, spatial domain, frequency domain

## Abstract

Image-based computer-aided diagnosis (CAD) systems have been developed to assist doctors in the diagnosis of thyroid cancer using ultrasound thyroid images. However, the performance of these systems is strongly dependent on the selection of detection and classification methods. Although there are previous researches on this topic, there is still room for enhancement of the classification accuracy of the existing methods. To address this issue, we propose an artificial intelligence-based method for enhancing the performance of the thyroid nodule classification system. Thus, we extract image features from ultrasound thyroid images in two domains: spatial domain based on deep learning, and frequency domain based on Fast Fourier transform (FFT). Using the extracted features, we perform a cascade classifier scheme for classifying the input thyroid images into either benign (negative) or malign (positive) cases. Through expensive experiments using a public dataset, the thyroid digital image database (TDID) dataset, we show that our proposed method outperforms the state-of-the-art methods and produces up-to-date classification results for the thyroid nodule classification problem.

## 1. Introduction

The traditional diagnostic technique, based on the expert knowledge of doctors, has a critical limitation in that the result of the diagnosis is heavily dependent on the personal knowledge and experience of the doctor. Consequently, the performance of diagnosis is limited and varies with the doctor’s experience. To surmount this limitation, a double screening scheme has been applied in some hospitals by employing an additional expert [1]. However, this scheme is expensive and time consuming. The development of artificial intelligence [2,3] and imaging techniques (such as magnetic resonance imaging (MRI), X-ray, and ultrasound) in medical research has led to the development of an image-based computer-aided diagnosis system (CAD) [4,5], which is widely used to assist doctors in the diagnosis of various kinds of diseases. The CAD system serves as the additional expert in the double screening scheme and makes suggestions to doctors during diagnosis of diseases. This diagnosis technique is based on captured images of several parts of the human body and a computer-based program for identifying abnormal signs in these parts in lieu of the professional knowledge of doctors. This technique has been successful in many applications, such as brain [6,7,8,9,10], breast [11,12,13,14,15,16,17], lung [18,19,20], and thyroid [21,22,23,24,25,26,27,28,29,30,31,32,33,34,35,36,37,38,39,40,41,42] nodule detection/classification problems. For diagnosing thyroid nodules, previous studies focused on designing computer-based systems to perform several functions to detect and/or classify thyroid images into several classes such as nodule versus non-nodule; benign versus malign; follicles versus fibrosis, etc. In our research, we focus on the classification of benign and malign cases of thyroid nodules.

Thyroid nodules are abnormal regions (lumps) that appear in the thyroid region of adult humans and may be an indicator of thyroid cancer [21,35]. Based on their characteristics, thyroid nodules are classified into two kinds: benign (negative or non-cancerous nodules that are not harmful to health) and malign (positive or cancerous nodules that can cause health problems). Fortunately, most detected thyroid nodules are benign [22]. However, with the appearance of thyroid nodules, patients may be confronted with various health or aesthetic problems. For example, large thyroid nodules, both benign and malign, may be visible and/or make it difficult for patients to breathe and swallow. More critically, malign thyroid nodules can produce an additional hormone called thyroxine, which causes some critical problems with patient’s health, including unexplained weight loss, tremors, and rapid heartbeat, and can also cause thyroid cancer. According to a report by the American Cancer Society, there were about 1950 patients estimated to have died among 62,450 new cases of thyroid cancer in 2015 [21]. However, the mortality rate can be reduced with early detection and treatment. Furthermore, determining whether a thyroid nodule is benign or malign is a hard task for doctors when it is based only on symptoms or experience. Consequently, detection and classification methods for thyroid nodules are critical for diagnosing problems with the thyroid.

For thyroid nodule diagnosis, thyroid imaging reporting and data system (TI-RADS) is referred to as several risk stratification systems for thyroid lesions, and TI-RADS scores using the ultrasound image have been adopted to indicate whether the patient has a problem with their thyroid [25]. In addition, methoxyisobutylisonitrile-single photon emission computed tomography (MIBI-SPECT) has been used for the diagnosis of thyroid nodules [43]. As the other method of thyroid nodule diagnosis, a non-surgical diagnosis method called fine needle inspiration (FNA) biopsy has been widely used [21,22,23,24]. However, because thyroid nodules are highly complex, about 10%–20% of thyroid nodule biopsies are non-diagnostic [22]. Furthermore, the main disadvantages of the FNA method are that it is labor-intensive, invasive, and costly, resulting in the CAD system becoming popular for assisting doctors in diagnosing thyroid nodules.

Previous studies on the thyroid nodule classification problem can be categorized into two groups: handcrafted-based and deep learning-based methods. In the first category, researchers used several traditional image feature extraction methods to extract efficient image features for the classification problem. In [26], the authors used up to 78 textural features, such as the gray level co-occurrence matrix, statistical features, the gray level run-length matrix, and Law’s texture energy measures, to describe an input ultrasound thyroid image. These features are then inputted into a support vector machine (SVM) to classify the input image into several categories, such as nodule versus non-nodule and follicles versus fibrosis. In [27], the authors showed the efficiency of a multi-scale features approach based on wavelet analysis for thyroid nodule classification. Based on the handcrafted image features, the authors in [31] analyzed the performance of linear and non-linear classifiers for ultrasound thyroid nodule images. They showed that the two kinds of classification methods yield similar (comparable) levels of classification accuracy. Based on the characteristics of thyroid nodules, Raghavendra et al. [35] found that ultrasound thyroid images are subjected to different threshold values to generate corresponding binary images. Based on this observation, they used segmentation-based fractal texture analysis (SFTA) method to extract image features from binarized images at different threshold values for the thyroid nodule classification problem. Furthermore, they combined the fractal texture features with spatial handcrafted texture features to enhance the performance of the classification system. Consequently, they showed that the handcrafted-based features are efficient for characterizing and categorizing thyroid nodules. However, the accuracy of this kind of methods is limited.

In the second category, deep learning-based methods, based on constructing a deep neural network for image classification, have been used to overcome the limitations of handcrafted-based methods. As explained in the preceding section, the handcrafted-based methods are based on pre-designed image feature extractors for extracting efficient image features and a classification method for classification. Thus, the performance of classification methods is dependent on the selection of image feature extractors, which usually affect only limited aspects of the thyroid nodule classification problem, and a classification method. By applying the deep learning-based method to the problem, these dependencies can be removed, and the feature extractors and classification methods are driven by training data. In methods of this type, researchers construct a deep learning-based model for various purposes, such as feature extraction, or the classification or detection of thyroid nodules in captured ultrasound images. In a study by Sundar et al. [32], they proposed a framework for ultrasound thyroid nodule image classification based on the use of a convolutional neural network (CNN). In their study, they first used a pre-trained CNN network as an image feature extractor to extract the image features of an inputted ultrasound thyroid nodule image. Based on the extracted image features, they used the SVM method to classify the input images into benign or malign classes. In their second study, they also constructed a new CNN network for image classification by either training from scratch or using a transfer learning technique (using VGG16-Net [44] or Inception-Net [45]). Similar to the study by Sundar et al., the study by Song et al. [34] also applied a transfer learning technique to successfully classify thyroid nodule images. Through their research, they demonstrated that CNN networks are suitable for thyroid nodule classification problems and produce good classification results. In recent studies by Song et al. [30] and Wang et al. [33], a detection and classification scheme was applied to the thyroid nodule classification problem. The rough position of possible nodule regions was first detected using either a multi-scale single-shot detection network (multi-scale SSD) or a YOLOv2 network. With the region detected, a fine classification step is performed to classify the nodule into a corresponding category, i.e., benign or malign. Although this approach can achieve high classification accuracy, it is time-consuming for nodule detection and requires a complex network design. In Table 1, we summarize our approach for ultrasound thyroid image classification.

As a hybrid approach of the methods just mentioned, a combination of deep and handcrafted image features is considered for the thyroid nodule classification problem. In this study, we propose a new method that combines the deep and handcrafted features with four novelties, as follows:

- First, we propose the use of information in the frequency domain for thyroid nodule image classification. In contrast to most of the previous studies, which only consider information on thyroid nodule images in the spatial domain, our study also explores the utility of information in the frequency domain for the thyroid nodule classification problem, using the Fast Fourier Transform (FFT) method, based on our observation of the characteristics of benign and malign nodules.
- Secondly, we propose a method for extracting the information in the frequency domain to differentiate between benign and malign cases. Based on the result of this proposed method, we construct a rule for classifying input images into one of three groups: *benign*, *benign-malign*, or *malign*, as explained in detail in subsequent sections.
- Thirdly, we propose a modified residual network (Resnet) by adopting global average pooling to summarize the feature maps of the last convolution layer, which reduces the number of network parameters, and by using batch normalization and dropout techniques to reduce the effects overfitting problem. Also, we combine the classification results using a deep learning-based method and a frequency-based method to enhance classification accuracy. For this purpose, we designed a cascade classifier classification system, based on the information extracted from the frequency and spatial domains.
- Finally, we make our algorithm available to the public through [46], so that other researchers can make fair assessments of our system.

In the rest of the paper, we first describe the proposed method for the thyroid nodule classification problem in Section 2. Using the proposed method, we perform various experiments using the TDID dataset to evaluate the performance of our proposed approach, as well as compare it to previous studies in the literature in Section 3. Finally, we give a conclusion of our study in Section 4.

## 2. Proposed Method

### 2.1. Overview of the Proposed Method

**Figure 1:**
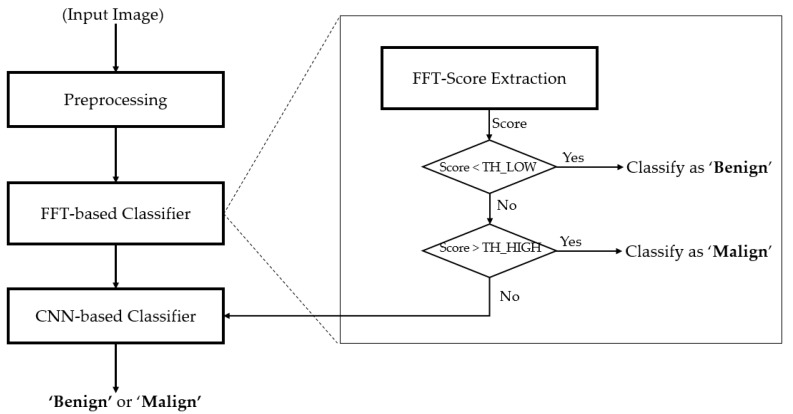
Main flow chart of our proposed method.

**Figure 2:**
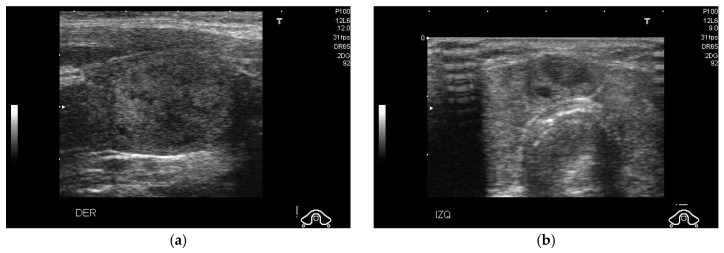
Example of captured ultrasound thyroid images: (**a**) a benign case, and (**b**) a malign case.

As explained in the preceding section, our study uses an ultrasound thyroid image to determine whether a patient has a thyroid problem. For this purpose, we propose an image-based classification method, as depicted in Figure 1. As shown in Figure 1, the captured ultrasound thyroid images are first preprocessed to extract useful image regions of interest (ROI), which contain information for our study, and reduce the effects of noise caused by background and artifact regions. This step is necessary because the captured ultrasound thyroid images may not only contain thyroid regions, as background regions and some additional artifact regions added by the capturing systems can be observed in Figure 2. Consequently, this kind of useless information can affect the performance of the classification system. This step is discussed in more detail in Section 2.2.

After the preprocessing step, our algorithm obtains a reduced thyroid ROI image that contains only the thyroid region and uses this ROI image for the classification steps. As shown in Figure 1, our study uses a cascade classifier based on Fast Fourier Transform (the FFT-based classifier in Figure 1) and a CNN (the CNN-based Classifier in Figure 1). For the first stage of classification, we use the FFT-based classifier to pre-classify the input thyroid images into one of three groups (*benign*, *benign-malign*, or *malign*) using two threshold values (TH_LOW and TH_HIGH, presented in Figure 1). The *benign* group indicates that the input image has been labeled as definitely benign. The *malign* group indicates that the input image has been labeled as definitely malign, while the *benign-malign* group denotes an undecided region of the input image using the FFT-based method. The value of TH_LOW and TH_HIGH is decided experimentally based on the training data and the distribution of benign and malign images in the frequency domain. A detailed description of the FFT-based method is given in Section 2.3.

In the second stage, we use the CNN-based method to further process the thyroid images if they are classified as *benign-malign* using the FFT-based method. Section 2.4 provides a detailed description of this stage.

### 2.2. Preprocessing of Captured Thyroid Images

In Figure 2, we present two examples of captured ultrasound thyroid images. As can be seen in Figure 2, a normal ultrasound thyroid image contains three different parts: background, artifact (additional text or indicator made by the capturing system), and thyroid region. Because our system is for classifying thyroid region images into benign (negative case) or malign (positive case), the background and artifact regions should be removed from the classification system to enhance classification accuracy.

**Figure 3:**
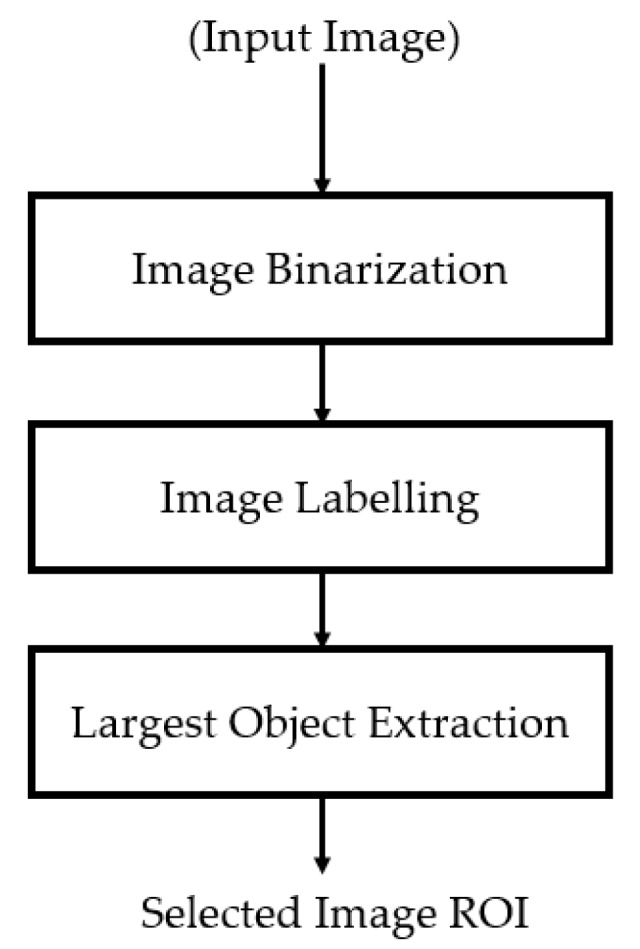
Pre-processing method for thyroid image ROI extraction.

**Figure 4:**
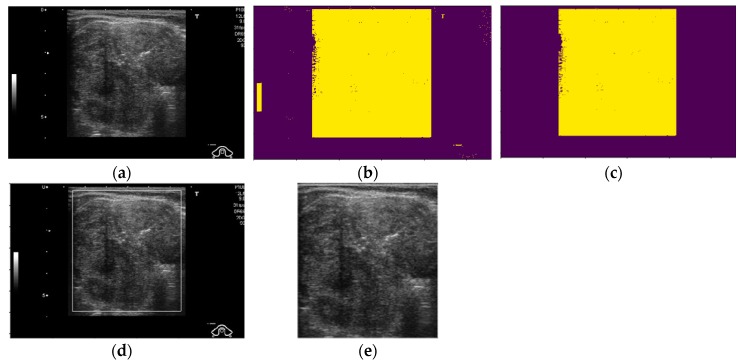
Sample result of our thyroid region ROI extraction method: (**a**) input captured ultrasound thyroid image with additional background and artifacts, (**b**) binarized image, (**c**) detection of largest object using labeling method, (**d**) final detected (localized) thyroid region, and (**e**) extracted thyroid region image.

As shown in Figure 2, the background region in ultrasound thyroid images normally has low illumination (close to zero), and the artifact has very high illumination (close to 255), while the thyroid region has a broader range of illumination (from 0 to 255 gray levels). Based on these characteristics, we preprocess the input ultrasound thyroid images using the method depicted in Figure 3 to extract only the thyroid region for our proposed method. Figure 4 presents visualized results of a sample implementation of these preprocessing steps. As shown in Figure 3, our study first performs an image binarization step to roughly obtain the thyroid region. Because of the ultrasound thyroid image characteristics mentioned earlier, the binarized image normally contains three parts: background with a gray level of zero, thyroid region with a gray level of one, and several additional small objects caused by the effects of artifacts on images. A sample result of this step is presented in Figure 4b using the input thyroid image in Figure 4a. As shown in Figure 4a, b, the thyroid region is the largest object in the binarized image. Based on this observation, next, we perform an image labeling step to find the largest object in the binarized image. Based on the image labeling, we can decide to retain the largest object and remove the smaller objects and background. Consequently, we can obtain the final thyroid region, as shown in Figure 4c. The final thyroid image in Figure 4e is obtained by combining the result of the thyroid region localization with the input image. This final image is then used as the input in the next step of our proposed method. In our study, we used the Otsu method, an adaptive thresholding method, for image binarization [47].

### 2.3. Classification in the Frequency Domain

**Figure 5:**
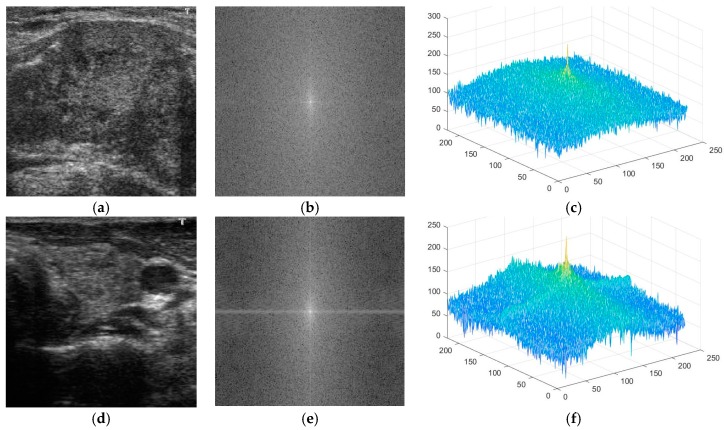
Examples of benign and malign cases in both spatial (**a**,**d**) and frequency (**b**,**c**) and (**e**,**f**) domains, respectively.

As explained in Section 1, the malign cases are usually differentiated from benign cases by the appearance of some small round-shaped areas with the calcification phenomenon [25]. This phenomenon causes an unusual change in the pixel values of ultrasound thyroid images of malign cases in contrast with normal (benign) cases. With the appearance of thyroid nodules, the distribution of frequency components in the frequency domain is different for benign and malign cases. In Figure 5, we show benign (Figure 5a) thyroid images in spatial domain, with the corresponding power spectrums (Figure 5b,c) in frequency domain. In addition, we show malign (Figure 5d) thyroid images in spatial domain, with the corresponding power spectrums (Figure 5e,f) in frequency domain. In contrast to spatial domain, which reflects the appearance of pixels by their intensities, the representation of the image in frequency domain reflects the amount of change in pixel intensities in the spatial domain [48]. These kinds of analyses in spatial and frequency domain images have been widely used in digital signal processing. As a result, the power spectrum images of Figure 5b,c,e,f represent the distribution of image energy over frequencies of entire images instead of local parts of Figure 5a,d. As shown in Figure 5, the appearance of the thyroid nodule with calcification phenomenon causes a change in pixel values in certain directions, such as horizontal and/or vertical direction faster (high frequency component) than in other directions, which show the brighter pixels in corresponding directions of Figure 5b,e.

It can be seen from this figure that the distribution of frequency components in a benign case seems to be a normal distribution (Gaussian-like distribution), with the center at the zero (center) frequency (the DC component), shown in Figure 5b,c. However, with the appearance of thyroid nodules and the calcification phenomenon, the distribution of frequency components is altered, as shown in Figure 5e,f. The appearance of thyroid nodules causes a change in pixel values in certain directions (horizontal or vertical direction) faster than in other directions. Consequently, the distribution of the frequency components is not in a Gaussian-like shape, but is altered to a weird shape shown in Figure 5f.

**Figure 6:**
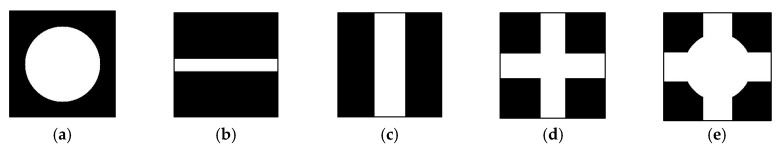
Shape of masks used in measuring the $fft_{score}$ in our study for (**a**) circle shape, (**b**) horizontal shape, (**c**) vertical shape, (**d**) vertical-horizontal (plus) shape, and (**e**) circle-plus shape

Based on this observation, in this study, we propose a new measurement method for distinguishing between benign and malign cases in the frequency domain. To measure the difference between the images of benign and malign cases, we propose the use of a ratio measurement between the selected frequency components over the entire frequency components. We defined $fft_{score}$ as Equation (1), in which *P* denotes the total power spectrum of an image, and *P_i_* denotes the total power spectrum inside the selected frequency region. Consequently, $fft_{score\;}\;$ of benign cases tend to be smaller than those of malign cases. Based on our observation and Figure 5, we used various shapes of masks (selected frequency components) in our experiments, as shown in Figure 6, including the circle (Figure 6a), horizontal (Figure 6b), vertical (Figure 6c), vertical-horizontal (plus) shape (Figure 6d), and circle-vertical-horizontal (circle-plus) shape (Figure 6e). The use of various shapes of selected frequency regions helps with exploring the characteristics of benign and malign cases in the frequency domain to obtain good classification results. The radius of the circle (Figure 6a,e), and the size of the horizontal and vertical bar (Figure 6b,e) are experimentally determined in our experiments using the training dataset.

(1) $$\;fft_{score}=\;\frac{P_{i}}{P}$$

Based on the above observation in the frequency domain of benign and malign cases, our study uses these characteristics to classify the thyroid images into three groups: *benign*, *benign-malign*, or *malign*, which stand for *definitely benign*, *fusion of benign-malign*, and *definitely malign*, respectively. As explained in Section 2.1, the *benign-malign* cases are further classified using the CNN-based method, while the *benign* and *malign* cases are used directly as the output for our proposed method.

### 2.4. CNN-Based Classification Method

For the second classifier used in our study, we use a deep learning-based method to further classify thyroid images into benign or malign classes if they are pre-classified as *benign-malign* by the FFT-based classifier. The deep learning method is a new and up-to-date method used in various signal processing fields such as computer vision [44,45,49,50].

**Figure 7:**
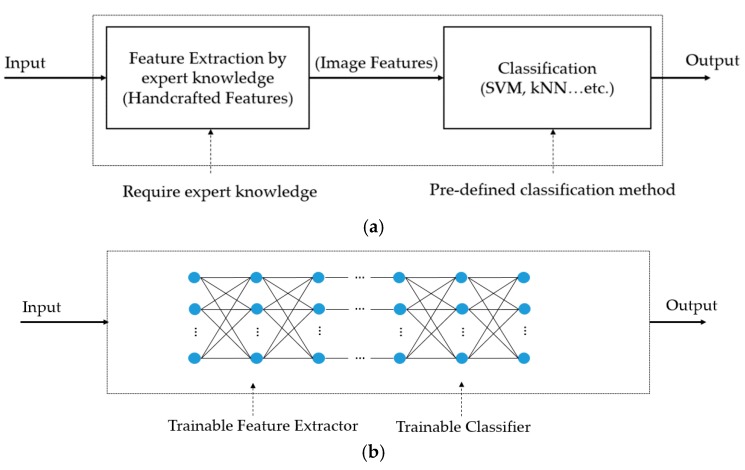
Difference between (**a**) conventional image classification method, and (**b**) deep learning-based classification method.

The convolutional neural network (CNN), a specific kind of deep learning method, is a popular deep learning framework and has been proved to be suitable for computer vision systems through its superior performance over conventional handcrafted-based methods. This kind of method has been successfully applied to many computer vision tasks, such as image classification [44,45,49,50], object detection [51,52], and image depth estimation [53]. Unlike conventional image-based classification methods, which are separated into two parts, (image feature extraction and image feature classification), as shown in Figure 7a, the CNN-based method constructs a concrete classification system by concatenating these two parts using the concept of the multi-layer perceptron (MLP) network with trainable network parameters (connection weights between neurons), as shown in Figure 7b. Because of this difference, the deep learning-based method is more flexible than conventional methods and has ability of learning from data that are limited in conventional methods.

**Figure 8:**
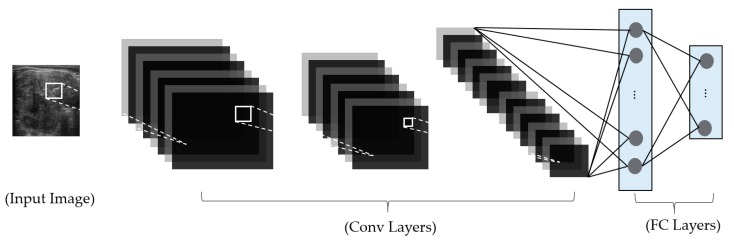
General structure of a CNN network for the image classification problem.

In Figure 8, we show the conventional structure of a CNN network used for an image-based classification system. As shown in this figure, a conventional CNN network contains two continuous parts: convolution layers (Conv) and fully-connected (dense) (FC) layers. These two parts are used to perform similar roles of feature extraction and classification in the conventional image classification methods in Figure 7a. The convolution layers use the convolution operation to extract the useful features from input images. The output of the convolution layers are the image’s features, which are then fed to the dense layers to perform the classification task. A distinctive characteristic of the CNN network is that it uses the convolution operation for image feature extraction. First, the use of the convolution operation helps *filter-out* the input images using trainable convolution kernels so we can select the useful features for each problem, instead of using the fixed feature extraction method. Secondly, the convolution operation performs a weight-sharing scheme in a conventional neural network. Consequently, it helps to significantly reduce the number of network parameters and make it possible to construct a deep network. Because of this design, the CNN network outperforms the conventional image-based classification methods. To reduce the effects of the overfitting problem of CNN, previous studies proposed many schemes, such as the dropout [54], data augmentation [49,55], and fine-tuning (transfer learning) techniques [24,28].

**Figure 9:**
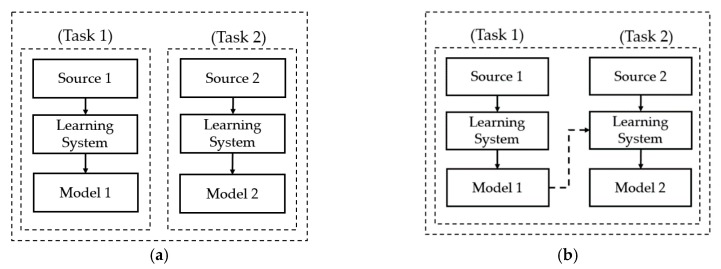
Demonstration of the difference between conventional training and transfer learning techniques (**a**) conventional learning method versus (**b**) transfer learning technique.

As explained in Section 1, one of the main problems with the medical image processing system is insufficient data for training (constructing) systems. Because of this problem, previous studies suggest the use of fine-tuning (transfer learning) techniques to successfully train a CNN network for medical image processing systems [24,28,32]. In our study, we also invoke this technique for the thyroid nodule classification problem. In Figure 9, we present a demonstration of the difference between conventional training and transfer learning techniques in a machine learning system [56]. The transfer learning technique is not new, but it has strong power in a machine learning-based system, especially for training a CNN network. This advantage is offered by its methodology, where we utilize some parts of an existing system in a new system. As shown in Figure 9a, the conventional training method trains a system using source data, without additional information on related problems. This training method makes the machine learning system fit with a single problem. However, the transfer learning method, as depicted in Figure 9b, trains a system using two sources, including source data on its own task, and additional knowledge sources on related problems. As we can see from Figure 9, the transfer learning technique trains a machine learning system using richer information than a conventional learning technique. Consequently, it can offer a superior performance to conventional learning techniques. Based on the transfer learning technique, we designed our CNN-based classification network using several existing pre-trained CNN networks, including Resnet18, Resnet34, and Resnet50 models [50]. The use of three different architectures of the CNN model in our experiments is to investigate the classification performance of the same network architecture at different depths. Although it is possible to use various alternative CNN structures such as VGG [44] and Inception [45], the use of Resnet models in our experiments is an exemplar for investigating the performance of our proposed method. These pre-trained CNN architectures and models were successfully trained using a large image classification dataset called Imagenet [50]. To use these architectures, we reused all convolutional layers for image feature extraction. Because these layers were successfully pre-trained, they are suitable for extracting image features.

However, the classification layers are not reused because these pre-trained models were designed for a 1000-class classification problem, which is different from our problem requiring a 2-class (benign and malign) classification problem. Because of this difference, we designed our CNN network, as shown in Table 2. In this table, the (m, n, k) denotes the output shape of the tensor (image) of the last convolution layer of a base-model, and #*param* indicates the total number of parameters of our CNN network reusing layers from the base network. The shape (m, n, k) is dependent on the base model. In our experiments, we use Resnet models (Resnet18, Resnet34, and Resnet50). Thus, the shape of the output of the last convolutional layer is (7, 7, 512) for Resnet18 and Resnet34, and (7, 7, 2048) for Resnet50. Furthermore, #*param* is 11,186,889 for the use of the Resnet18-based model, 21,302,473 for the use of the Resnet34-based model, and 23,587,712 for the use of the Resnet50-based model.

### 2.5. Summaries on Differences of Proposed CNN Network from Conventional Resnet Network

- We use two neurons at the output of the network, instead of 1000 neurons in the conventional Resnet network, to fit with our problem in which we classify the input image into two classes of benign and malign cases.
- We use global average pooling to summarize the feature maps of the last convolution layer. As a result, we reduce the number of network parameters. Also, we also use batch normalization and dropout techniques to reduce the effects overfitting problem that normally occurs in training deep CNN networks.

## 3. Experimental Results

In this Section, we present various experiments using our proposed method mentioned in Section 2 in comparison with various previous methods using the thyroid digital image database (TDID) dataset.

### 3.1. Dataset and Experimental Environment

To evaluate the performance of our proposed method and compare it with previous studies, we use TDID, a public thyroid nodule image dataset created by Universidad Nacional de Colombia [25]. In total, the TDID dataset contains ultrasound thyroid images of 298 patients. For each patient, one or more ultrasound thyroid images were captured and provided to users with information on nodule region localization and corresponding TI-RADS scores to indicate whether the patient has a problem with their thyroid. All the images are in RGB format with the shape of 560-by-360 pixels. As explained by the author of this dataset, they do not provide information of how many patients are operated on because of the “exclusion of thyroid cancer”. In addition, this dataset does not have information of MIBI-SPECT and FNA, either. Therefore, we used only the TI-RADS scores provided from this dataset in our experiments.

Based on its definition, the TI-RADS score takes one among six values {2, 3, 4a, 4b, 4c, 5} with scores of 2 and 3, indicating cases of benign and no suspicious ultrasound (US) features, respectively, while scores of 4a, 4b, 4c, and 5 indicate cases of one suspicious US features, two suspicious US features, three suspicious US features and five suspicious US features, respectively. Based on this definition, we consider the images with TI-RADS scores of 2 or 3 as the benign (negative) cases, while the images with TI-RADS scores of 4a, 4b, 4c, and 5 are the malign (positive) cases. However, the images with TI-RADS score of 2 and 3 do not provide other information of non-malignant thyroid pathologies, like Hashimoto and Graves’ disease patients. Consequently, the TDID dataset provides a total of 52 patients with benign cases and 246 patients with malign cases.

As explained in Section 2, our method requires a training dataset to train the classification model. Therefore, we split the entire TDID dataset into training and testing datasets for use in our experiments. Furthermore, we used a five-fold cross-validation method to validate the performance of our classification method. For the training dataset, the thyroid images of 41 patients among 52 patients with benign thyroid nodules, and 196 patients among the 246 patients with malign thyroid nodules were used, and, for the testing data, the images of the other 11 patients with benign cases and 50 patients with malign cases were used. We train our classification model using the training dataset, and this trained model is validated, and its performance is measured using the testing dataset. In Table 3, we present a description of the TDID dataset and its training/testing sub-dataset used in our experiments.

**Figure 10:**
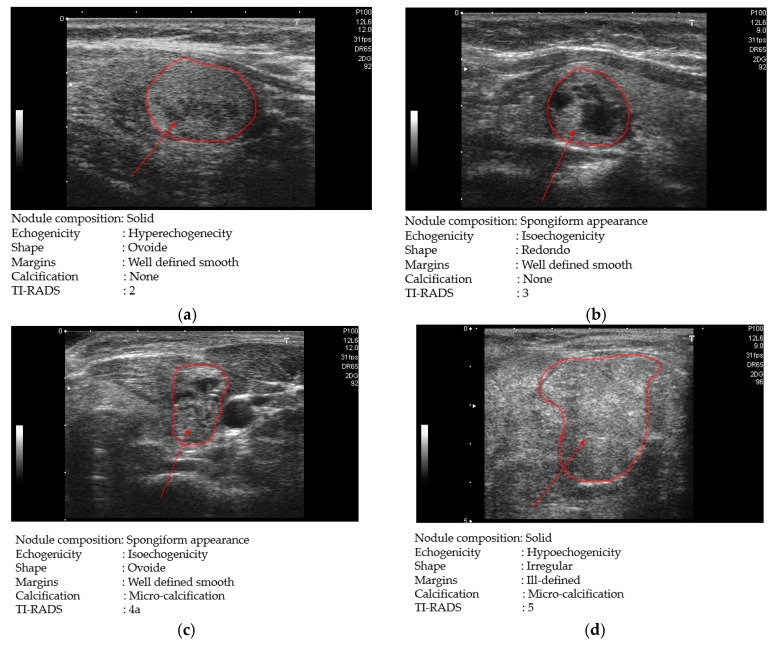
Examples of ultrasound thyroid images in TDID dataset: (**a**) a benign case image with thyroid imaging reporting and data system (TI-RADS) score of 2; (**b**) a benign case image with TI-RADS score of 3; (**c**) a malign case image with TI-RADS score of 4; and (**d**) a malign case image with TI-RADS score of 5.

In Figure 10, we show some examples of ultrasound thyroid images in the TDID dataset. Figure 10a,b shows two benign case images with TI-RADS scores of 2 and 3, respectively; while Figure 10c,d shows two other malign case images with TI-RADS scores of 4 and 5, respectively. As shown in Figure 10c,d, the malign case images contain nodules with calcification phenomenon (round shape of texture with spotted white regions). Differing from these two case images, the benign case image in Figure 10a does not contain these features and it seems to be easily distinguished from the malign cases of Figure 10c,d. However, the benign case image with the TI-RADS score of 3 (Figure 10b) contains some similar texture features to the malign case image in Figure 10c,d such as round shapes of texture features, high illumination pixels, etc. As a result, this kind of benign case image is hard to distinguish from malign case images.

In this experiment, we used a desktop computer with an Intel Core i7 central processing unit (CPU) (Intel Corporation, Santa Clara, CA, USA) and 64 GB of RAM. For the training and testing of proposed algorithm, we used a TitanX GPU card [57] and Tensorflow library [58].

### 3.2. Training of CNN Model

In our first experiment, we performed fine-tuning on the CNN models, as mentioned in Section 2.4, for the thyroid nodule classification problem. As explained in Section 2.4, we used three different CNN base architectures—Resnet18, Resnet34, and Resnet50—to construct our CNN network for the purpose of investigating the effects of network depth on classification performance. Based on the transfer learning technique, we set a small initial learning rate (0.0001), and the number of epochs for the training process was set to 10 epochs. Furthermore, we continuously reduce the learning rate after every epoch, as shown in Table 4.

**Figure 11:**
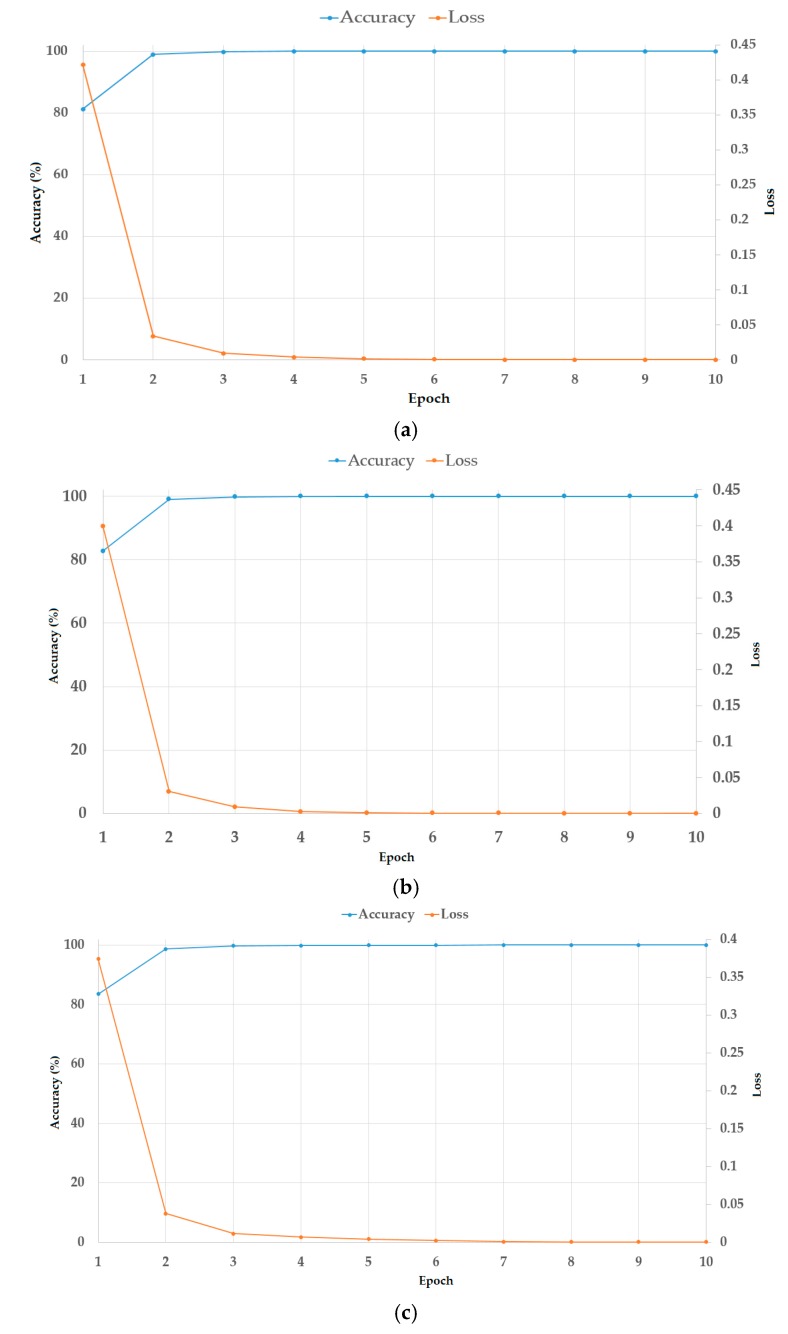
Training loss and accuracy of the CNN model in our experiments: (**a**) using a Resnet18-based network, (**b**) using a Resnet34-based network, and (**c**) using a Resnet50-based network

To train the CNN networks, we used the training data presented in Table 3 (including the data on the 41 patients with benign thyroid nodules and 196 patients with malign thyroid nodules patients) five times to perform a 5-fold cross-validation scheme. The number of training data are randomly selected from the entire TDID dataset. Through the training process, we obtain CNN models for benign/malign classification problems. In Figure 11, we present the results of our training process using the training dataset. Figure 11 shows the change in classification accuracy and loss with increments in training epochs. From this figure, it can be seen that training accuracy increases toward 100%, and the loss decreases toward 0, in all three tests with Resnet18-based CNN network, Resnet34-based CNN network, and Resnet50-based CNN network, respectively. This result indicates that the training procedure was successfully done in our experiments.

### 3.3. Criteria for Performance Measurement

To measure the performance of a medical image-based classification method, our study uses three matrices—accuracy, specificity, and sensitivity—which have been widely used in previous studies [24,28,32,59]. As mentioned in the previous sections, our study deals with the thyroid nodule classification problem, in which an input thyroid image is classified into two classes of benign (negative case) or malign (positive case). Consequently, sensitivity is defined as the ability of a classification method to correctly identify a person who has a disease (malign nodule), and specificity is defined as the ability of a classification method to correctly identify a person who does not have a disease (benign nodule). Based on this definition, the sensitivity is measured as the proportion of patients who have positive test results (correctly classified as malign) over the total number of patients with malignant cases, and the specificity is measured as the proportion of patients who have negative test results (correctly classified as benign) over the total number of patients with benign cases. Finally, the overall accuracy of the classification method is measured by taking the proportion of correct classification results over the total number of tests.

**Figure 12:**
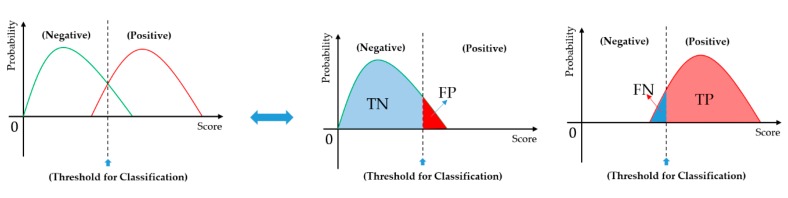
Demonstration of possible outcomes of a classification system.

Suppose we have a test dataset with a total of *m* samples in which there are *n* positive (malign) and *p* negative (benign) samples. Through a classification system, the outcomes are four possible cases: true negative (*TN*), false positive (*FP*), true positive (*TP*), and false negative (*FN*). *TN* is when the proportion of negative (benign) cases are correctly classified as negative cases. *TP* is when the proportion of positive (malign) cases are correctly classified as positive cases. These two possible outcomes are the correct classification results of a classification system. Therefore, high values of *TN* and *TP* indicate high performance of the classification system. The other two possible outcomes of *FN* and *FP* are the error cases of the classification system. *FP* is when a negative sample is incorrectly classified as a positive one, and *FN* is the case when a positive sample is incorrectly classified as a negative one. Based on these definitions, we can obtain two equations: *TN* + *FP* = *n* and *TP* + *FN* = *p*. In Figure 12, we present a demonstration of the meaning of these possible outcomes. Based on these definitions, accuracy, specificity, and sensitivity measurements are defined as Equations (2)–(4). It can be seen from these equations that a high-performance classification system tends to have high values of accuracy, specificity, and sensitivity. Furthermore, the specificity indicates the proficiency of the classification system in detecting negative (benign) cases, while the sensitivity indicates the proficiency of the classification system in detecting positive (malign) cases.

(2) $$Accuracy=\;\frac{TP+TN}{TP+TN+FP+FN}$$

(3) $$Specificity=\;\frac{TN}{TN+\;FP}$$

(4) $$Sensitivity=\;\frac{TP}{TP+\;FN}$$

### 3.4. Testing of Thyroid Nodule Classification

#### 3.4.1. Performance Comparisons of CNN with or without Frequency Information

With the trained CNN models (based on Resnet18, Resnet34, and Resnet50) obtained using the training procedure outlined in Section 3.2, we measured the performance of the CNN-based classifier using the test dataset. As shown in Table 3, the test dataset contains ultrasound thyroid images of 61 patients, among whom 11 patients have benign cases, and 50 patients have malign cases. The detailed experimental results are presented in Table 5. In this table, we measured classification performance in terms of sensitivity, specificity, and accuracy, as shown in Equations (2)–(4). The Resnet18-based CNN network produced an overall accuracy of 82.983%, with sensitivity at 84.78% and specificity at 72.401%. The overall classification accuracy is enhanced by using deeper CNN network architectures, with an accuracy of 84.383% using the Resnet34-based CNN network and 87.131% using the Resnet50-based CNN network. Along with the increment in overall accuracy, sensitivity also increases to 84.478% using the Resnet18-based CNN network, 85.535% using the Resnet34-based CNN network, and 90.597% using the Resnet50-based network. However, the change in specificity is dissimilar from the changes in sensitivity and overall accuracy. As shown in Table 5, we obtained the largest specificity, of 76.037%, using the Resnet34-based CNN network, and the smallest specificity, 63.741%, using the Resnet50-based CNN network. Using the shallowest CNN network, the Resnet18-based network, we obtained a specificity of 72.401%. This result indicates that the deeper network is useful for enhancing the classification performance in terms of overall accuracy and sensitivity, but is not sufficient to enhance specificity. In other words, the use of deeper networks is adequate for classifying the malign cases, while the shallower network is adequate for classifying the benign cases. Through this experimental result, we confirm that the CNN-based network is suitable for classifying ultrasound thyroid images with an overall accuracy of close to 87% using the TDID dataset.

In our next experiments, we performed further experiments using our proposed method by cascading the classification, based on the FFT method and the CNN methods explained in Section 2. In these experiments, we tested our proposed method using five different masks for extracting information in the frequency domains, as explained in Section 2.3 and Figure 6, including circle, horizontal, vertical, plus, and circle-plus masks. The use of these different masks is to explore the most suitable frequency components useful for our classification problem. The detailed experimental results are presented in Table 6, Table 7, Table 8, Table 9 and Table 10 for the different masks.

With the circle mask (Figure 6a), we obtained an overall accuracy of 86.091%, 88.017%, and 90.883% using the Resnet18-based CNN network, Resnet34-based CNN network, and Resnet50-based CNN network. These overall accuracies are much higher than those of 82.983%, 84.383%, and 87.131%, presented in Table 5. This result confirms that our proposed method using the circle mask is sufficient to enhance the classification accuracy of the thyroid nodule classification system. As shown in Table 5 and Table 6, the increment in overall accuracy is caused by the fact that our proposed method helps to increase the sensitivity, and it does not help enhance the specificity measurement. Essentially, our proposed method enhances classification performance for malign cases using the TDID dataset. The reason is that the TDID dataset contains more malign cases than benign cases, as shown in Table 3. Consequently, the classification in the frequency domain is focused primarily on malign cases rather than benign cases. In our future research, we will investigate the classification performance with a larger dataset. However, it is presently very difficult to obtain a dataset for experiments, because it takes time and requires the consent of the patients on account of the personal information involved.

Similar to the case of the circle mask, Table 7, Table 8, Table 9 and Table 10 present our experimental results for the other four mask shapes of Figure 6. As shown in these tables, all these masks can help enhance the classification accuracy of a classification system. Among the four masks, the circle-plus mask (Figure 6e) produced the highest classification accuracy, with an overall accuracy of 87.194%, 88.901%, and 90.616% for three CNN-based networks: the Resnet18-based CNN network, Resnet34-based CNN network, and Resnet50-based CNN network, respectively. Compared with the experimental results in Table 6, we can see that the classification accuracy obtained with the use of the circle-plus mask is comparable with using the circle mask. Through these experiments, we confirm that the use of image information extracted in the frequency domain is sufficient for enhancing the performance of thyroid nodule classification systems using the TDID dataset. Furthermore, we see that the circle mask can be selected for extracting image features in the frequency domain using Equation (1) among various possible mask shapes presented in Figure 6.

#### 3.4.2. Performance Comparisons of Proposed Method with the State-of-the Art Methods

As explained in Section 1, the thyroid nodule classification systems were successfully implemented using a CNN network with or without transfer learning technique. In the study by Zhu et al. [24], they used the Resnet18-based CNN network and transfer learning technique for the thyroid nodule classification problem. In the results of this study, they obtained an overall accuracy of about 84% using the TDID dataset. Similarly, in the study by Chi et al. [28], they used a different CNN network, called GoogLeNet [45], and transfer learning technique for the classification problem. More broadly, Sundar et al. [32] performed various experiments based on CNN networks, such as training from scratch, fine-tuning, and feature extraction based on pre-trained CNN networks followed by an SVM classification. Using a combined dataset of TDID and a private dataset, they achieved a very good classification accuracy. They obtained an overall accuracy of about 82% using a CNN network without the transfer learning technique, and an overall accuracy of up to 89% with the transfer learning technique. However, their experiments were performed using a large dataset that is a combination of TDID and a private dataset. Therefore, the distribution of benign and malign was better than with the use of the TDID dataset alone, and this yielded better results than using a single dataset. As shown in these studies, the CNN network with the transfer learning technique yielded a better classification performance compared to other network architectures. However, as explained in Section 1, using the CNN network can only extract information on texture features in the spatial domain. Furthermore, as shown in the studies mentioned, the experiments were not performed with the TDID dataset alone. Therefore, a comparison with our study would be imbalanced. For a fair comparison with our study, we performed the experiment in Section 3.4.1 using a similar system setting for network architecture and dataset (using a CNN network with transfer learning on the same dataset). A detailed comparison and the classification accuracies are given in Table 11.

Although the overall accuracies reported in [24] are 93.75%, this was obtained by performing the data augmentation on both training and testing datasets, which is different to our experiments. For comparison purpose, we compare our classification accuracy with the accuracy obtained by [24] using the original TDID dataset. As reported in [24], they obtained a classification accuracy of about 84.0% using Resnet18-based network with the TDID dataset. In addition, the studies by Chi et al. [28], and Sundar et al. [32] did not perform experiments using the TDID dataset only, but a fusion of some datasets. For a fair comparison, we also performed experiments using their proposed methods with the same training and testing datasets with our experiments. In detail, we obtain an overall classification accuracy 79.36% using the GoogLeNet-based CNN network, and 77.57% using VGG16-Net-based CNN network. As shown in Section 3.4.1 and Table 11, our proposed method outperformed these previous studies by yielding an overall accuracy of up to 90.88%. Through these experiments, we confirm that frequency information is sufficient for enhancing the classification performance of a thyroid nodule classification system, and our proposed method outperformed previous studies using the TDID dataset. Furthermore, the deep network is more adequate than the shallow network for the classification problem.

### 3.5. Analysis and Discussion

**Figure 13:**
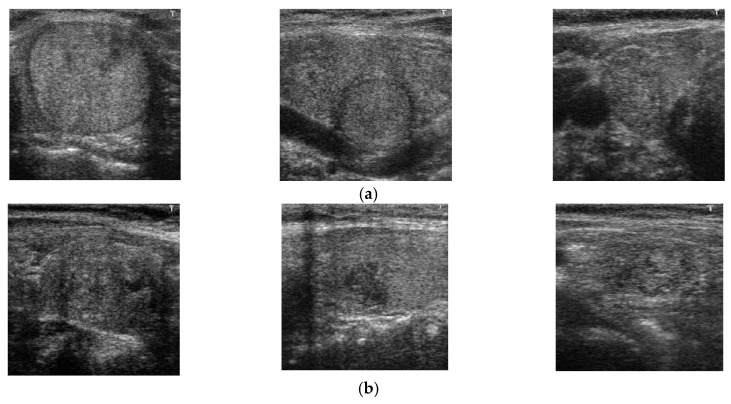
Examples of correct classification result using our proposed method using (**a**) benign case images which are similar to malign case images; and (**b**) malign case images which are similar to benign case images.

To demonstrate the efficiency of our proposed method, Figure 13 shows some examples of correct classification results using our proposed method. In this figure, Figure 13a shows some classification results of benign case images and Figure 13b shows some classification results of malign case images. The benign case images in Figure 13a seem to contain nodules by the appearance of round-shaped textures of different sizes. As a result, they are hard to recognize as benign case images by human perception, based on experience. However, they were correctly classified as benign case images by our proposed method. In contrast to Figure 13a, b presents some ground-truth malign case images. As shown in this figure, it is hard to recognize these images as malign case images by human perception, because the appearance of nodules is not clear, due to the small size of the nodules. By inputting these images to our proposed method, we correctly recognized them as malign case images. Based on this result, we conclude that our proposed method is sufficient for classifying ultrasound thyroid images into benign and malign classes. Also, it can provide valuable suggestions to medical doctors in diagnosing thyroid nodules using ultrasound images.

**Figure 14:**
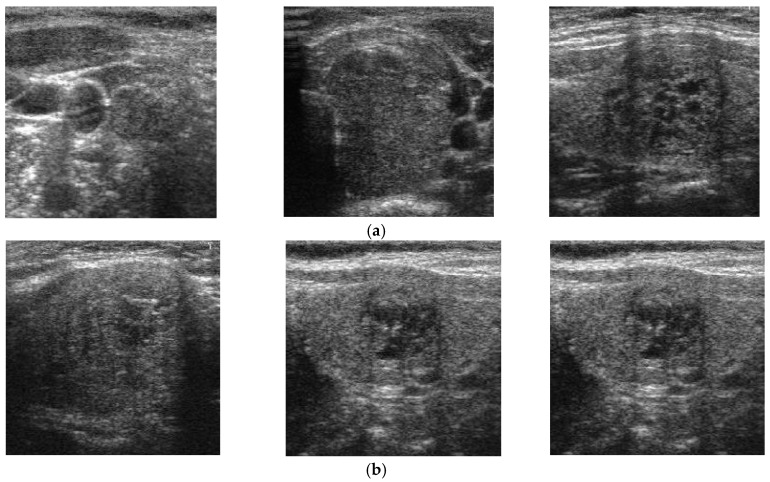
Example of error cases using our proposed method with the TDID dataset (**a**) benign-to-malign error cases, and (**b**) malign-to-benign error cases.

In Figure 14, we presented some examples of error cases obtained with our proposed method using the TDID dataset. Figure 14a shows some errors for a benign case, in which the proposed method incorrectly classified a benign case as malign, and Figure 14b shows errors for a malign case in which the proposed method incorrectly classified a malign case as a benign case. As shown in Figure 14a, although the images are benign, they contain several features that normally appear in the malign cases, such as the circle shape nodules, with a high illumination that makes them look like the malign nodule with calcification phenomenon. Consequently, they were incorrectly classified as malign. In Figure 14b, although the images are malign cases, the nodules are small and do not appear clearly distinguishable from benign cases. Due to this problem, they were incorrectly classified as benign cases. Through this example and the experimental results of our study and previous studies, we can see that the primary problem with the thyroid nodule classification system is data for training the classification model. As shown in previous studies [28,32], the CNN-based classification method works well when the networks were trained using a large dataset (combination of several datasets). Although our proposed method produced better classification results than previous studies with the same dataset (TDID dataset), its performance can be better if it is trained using a large dataset. In our future research, we will investigate this condition using a large dataset.

To demonstrate the performance of our proposed method in terms of processing speed, we measured the processing of our proposed method, and the experimental results are given in Table 12. The experimental environments are explained in Section 3.1. As shown in Table 12, it takes about 11.646 *milliseconds* to extract the thyroid ROI region in our proposed method. For the classification step, it takes about 5.093 *milliseconds* using the FFT-based method, and 17.525 *milliseconds* using our deep learning-based method. As explained in Section 2, we used a cascade classifier approach in our proposed method. Therefore, our proposed method takes at least 16.739 *milliseconds* (11.646 + 5.093) or, at most, 34.264 *milliseconds* (11.646 + 5.093 + 17.525) to classify an input ultrasound thyroid image into benign or malign classes. In other words, our proposed method can *process* data with a speed of 29 frames per second (fps) to about 60 (fps), according to the complexity of the input image. On average, our proposed method processes data at a speed of about 44 (fps).

**Figure 15:**
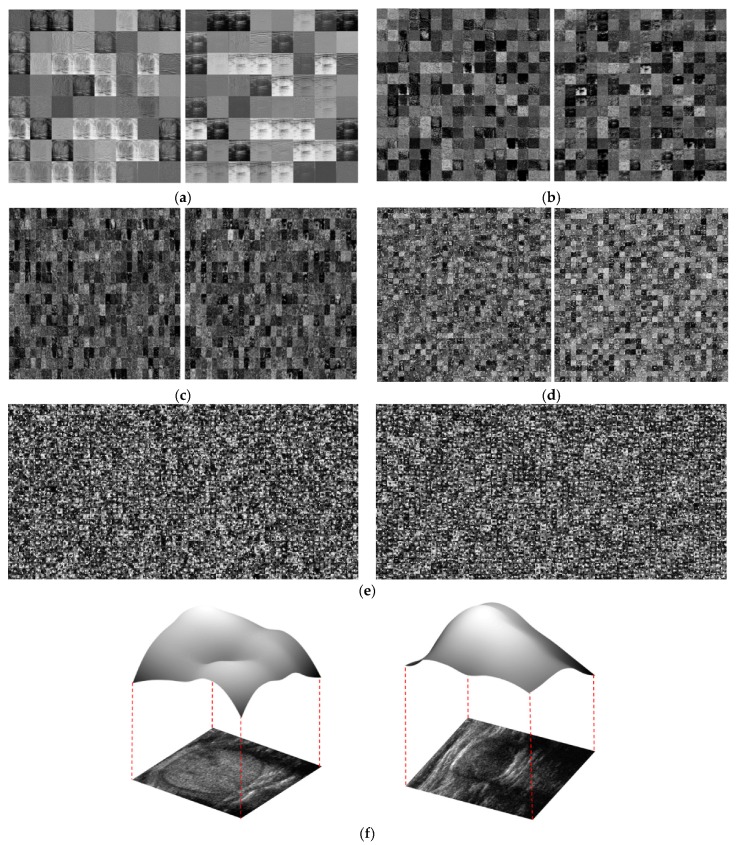
Example of feature maps obtained at the output of convolution blocks using our CNN architecture: From top to bottom: the output of (**a**) the first convolution layer; (**b**) the 10th convolution layer; (**c**) the 22nd convolution layer; (**d**) the 40th convolution layer; (**e**) the 49th convolution layer; and (**f**) average feature maps of (**e**). Left and right images of (**a**–**f**) show benign and malign case images, respectively.

For the next analysis, we extracted feature maps at several convolution layers of our CNN model to exploit the ability of the CNN network in learning texture features from the input image. The result of this experiment is presented in Figure 15 using a benign and malign case image in Figure 15f. All the left images of Figure 15a–f are the pictures of a feature map extracted from a convolutional neural network (CNN) with a benign image. The original input of the benign image to the CNN is shown at the bottom of the left image of Figure 15f. All the right images of Figure 15a–f are the pictures of a feature map from CNN with a malign image. The original input of the malign image to the CNN is also shown at the bottom of the right image of Figure 15f.

As shown in this figure, we extracted various feature maps according to the increment of the depth of the network: the 1st, 10th, 22nd, 40th, and 49th layer. The reason for choosing these layers is because they are the outputs of the first and the repeated convolution blocks in our model. As shown in Figure 15a–e, the feature maps came to be more abstracted as the depth of network increased. As the final output of the network, the feature maps in Figure 15e only contain abstract features with the size of 7-by-7 pixels. To make an intuitive observation of these final outputs, Figure 15f presents the corresponding three-dimensional feature maps obtained by averaging feature maps in Figure 15e for each case of benign and malign case images. It can be observed from these images that the extracted feature maps inherit differences between benign and malign case images. For the malign case image (on the right side of Figure 15f), the averaged feature map focuses on the thyroid nodule region by presenting higher feature magnitudes than other regions. For the case of the benign case image (on the left side of Figure 15f), the averaged feature map focuses on several regions on the input image, rather than a small region, as in the malign case image. Through this observation, we can conclude that our CNN-based network is efficient for extracting texture features for the thyroid nodule classification problem.

## 4. Conclusions

In this study, we proposed a thyroid nodule classification method using a cascade classifier scheme, based on the extracted information in both the spatial and frequency domains of an ultrasound thyroid image. Our proposed method processes an ultrasound image of the thyroid region to discriminate benign and malign cases in thyroid nodules. For this purpose, we first applied a classifier for the classification problem using image features in the frequency domain, which has not been done in previous studies. Secondly, the ambiguous samples with the first classifier are then further classified using a deep learning-based method. Through our expensive experiments, we show that our proposed method achieves a higher classification accuracy than various previous studies using the TDID dataset. Our algorithm is designed to provide more accurate suggestion to doctors (radiologists) in diagnosing thyroid nodules and reduce the number of operations on benign thyroid nodules. As a result, it is applicable in hospitals in daily routine. To use our algorithm requires a conventional desktop computer with a monitor and a commercial graphics processing unit (GPU) card for running deep neural networks, which costs less than $1500.

As future work, we would apply our algorithm in real applications, with the help of doctors, to enhance the performance of our algorithm, and make it applicable with the daily routine in hospitals. In addition, we would expand our work to include non-malignant thyroid pathologies, like Hashimoto and Graves’ disease patients, by collecting more data with the help of doctors in hospitals.

## Figures and Tables

**Table 1:** Summary of proposed study on the ultrasound thyroid nodule classification.

Category	Method	Strength	Weakness
Fusion of deep learning- and handcrafted-based methods	Uses information from both frequency and spatial domains for classification Uses a deep learning method to extract texture information Uses cascade classifiers to combine the two methods. (Our proposed method)	Analyzes the problem in two domains, i.e., frequency and spatial domains. Uses a cascade classifier scheme to enhance classification accuracy	More complicated than using only a single method (Fast Fourier transform (FFT)-based or convolutional neural network (CNN)-based methods)

**Table 2:** Architecture of the CNN network used in our experiments.

Layer	Input Shape	Output Shape	Number of Parameters
Base-Model	(224, 224, 3)	(m, n, k)	*#param*
Global Average Pooling	(m, n, k)	k	0
Batch Normalization	k	k	K × 4
Dropout	k	k	0
Output Layer (Dense layer)	k	2	2 × (k + 1)

**Table 3:** Description of the thyroid digital image database (TDID) dataset used in our experiments.

Benign Cases		Malign Cases		Total
Train Data	Test Data	Train Data	Test Data	
41 (patients)	11 (patients)	196 (patients)	50 (patients)	298 (patients)

**Table 4:** Learning rate schedule for training the CNN model in our experiments.

Initial Learning Rate	End Learning Rate	Learning Rate Reduction Rule	Number of Epochs
0.0001	0.00001	Continuous	10

**Table 5:** Performance of classification methods using various CNN architectures without frequency information (unit: %).

CNN Architecture	Sensitivity	Specificity	Accuracy
Resnet18-based Architecture	84.478	72.401	82.983
Resnet34-based Architecture	85.535	76.037	84.383
Resnet50-based Architecture	90.597	63.741	87.131

**Table 6:** Performance of our proposed method using various CNN architectures with frequency information based on circle mask (unit: %).

CNN Architecture	Sensitivity	Specificity	Accuracy
Resnet18-based Architecture	88.087	72.401	86.091
Resnet34-based Architecture	89.749	76.037	88.017
Resnet50-based Architecture (Proposed method)	94.933	63.741	90.883

**Table 7:** Performance of our proposed method using various CNN architectures with frequency information based on horizontal mask (unit: %).

CNN Architecture	Sensitivity	Specificity	Accuracy
Resnet18-based Architecture	85.195	72.401	83.608
Resnet34-based Architecture	86.791	76.037	85.472
Resnet50-based Architecture	90.818	63.741	87.321

**Table 8:** Performance of our proposed method using various CNN architectures with frequency information based on vertical mask (unit: %).

CNN Architecture	Sensitivity	Specificity	Accuracy
Resnet18-based Architecture	87.295	72.401	85.404
Resnet34-based Architecture	89.781	76.037	88.039
Resnet50-based Architecture	92.852	63.741	89.080

**Table 9:** Performance of our proposed method using various CNN architectures with frequency information based on vertical-horizontal mask (plus mask) (unit: %).

CNN Architecture	Sensitivity	Specificity	Accuracy
Resnet18-based Architecture	89.108	72.401	86.974
Resnet34-based Architecture	91.093	76.037	89.175
Resnet50-based Architecture	93.865	63.741	89.956

**Table 10:** Performance of our proposed method using various CNN architectures with frequency information based on circle-plus mask (unit: %).

CNN Architecture	Sensitivity	Specificity	Accuracy
Resnet18-based Architecture	89.361	72.401	87.194
Resnet34-based Architecture	90.775	76.037	88.901
Resnet50-based Architecture	94.625	63.741	90.616

**Table 11:** Comparison of the overall accuracy of previous studies and our proposed method with the TDID dataset (unit: %).

Methods		Accuracy
Zhu et al. [24]		84
Chi et al. [28]		79.36
Sundar et al. [32]	VGG16	77.57
	GoogLeNet	79.36
Proposed Method (using circle mask)		90.88

**Table 12:** Processing time of our proposed method (unit: millisecond).

Pre-Processing Step	FFT-Based Classification	Deep Learning-Based Classification	Total
11.646	5.093	17.525	34.264
